# Supplementary material for: Optimal minimal residual disease threshold in pediatric acute myeloid leukemia: A retrospective cohort study based on the TARGET database
Source: PLoS Med. 2026 May 8;23(5):e1005088. doi: 10.1371/journal.pmed.1005088 (PMC13155632; doi:10.1371/journal.pmed.1005088)
Supplement: S1 Code — (ZIP) [file pmed.1005088.s002.zip › S2 code/PROJ8_16_tbl2/PROJ8_16_tbl2_seg1_0.pdf]

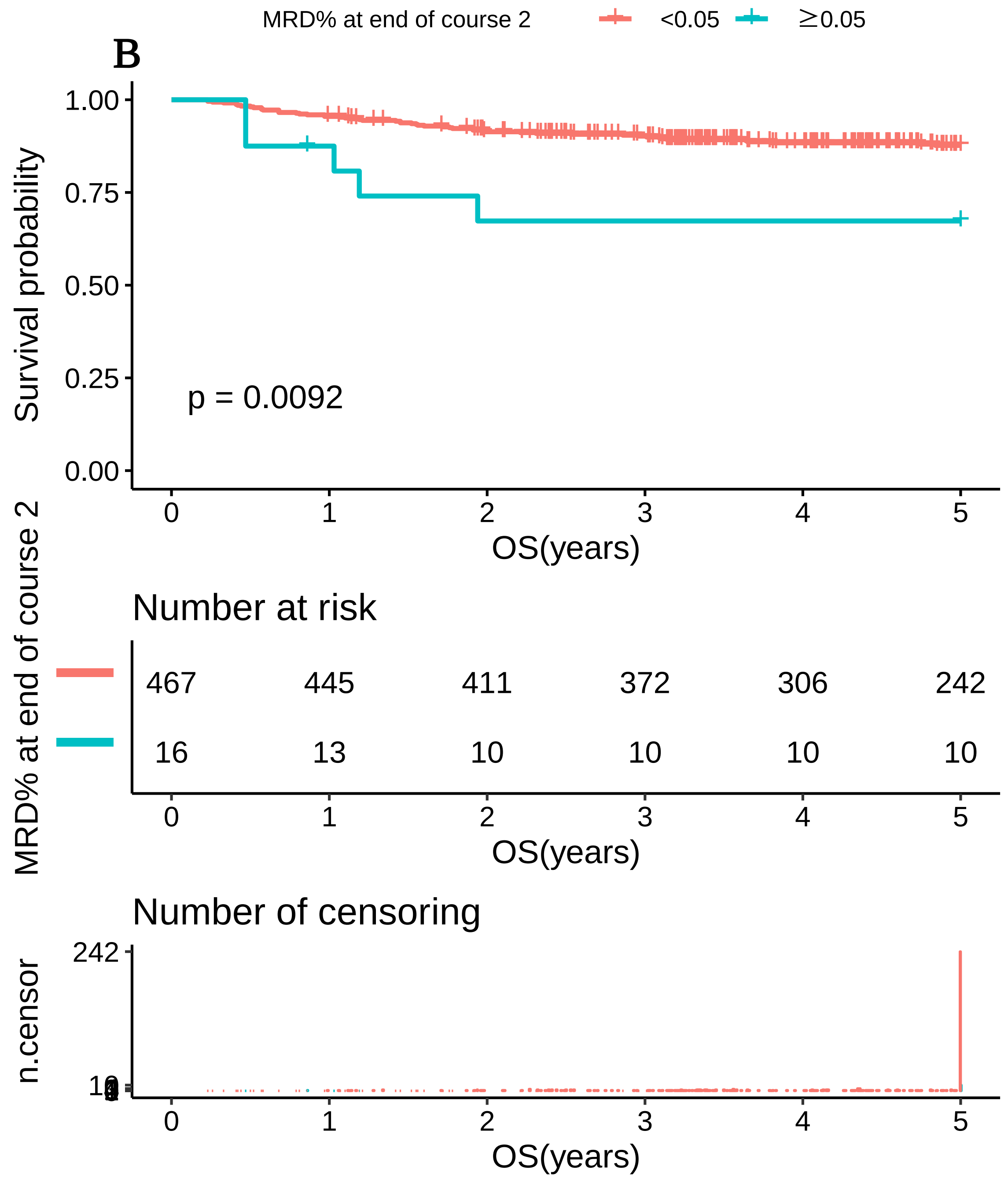

MRD% at end of course 2
<0.05
≥0.05
B
Survival probability
1.00
0.75
0.50
0.25
0.00
p = 0.0092
0
1
2
3
4
5
OS(years)
Number at risk
MRD% at end of course 2
467
445
411
372
306
242
16
13
10
10
10
10
Number of censoring
n.censor
242
OS(years)
